# Characterization and prognostic significance of alternative splicing events in lower‐grade diffuse gliomas

**DOI:** 10.1111/jcmm.15924

**Published:** 2020-10-02

**Authors:** Zheng Zhao, Guan‐Zhang Li, Yu‐Qing Liu, Ruo‐Yu Huang, Kuan‐Yu Wang, Hao‐Yu Jiang, Ren‐Peng Li, Rui‐Chao Chai, Chuan‐Bao Zhang, Fan Wu

**Affiliations:** ^1^ Department of Molecular Neuropathology Beijing Neurosurgical Institute Capital Medical University Beijing China; ^2^ Department of Neurosurgery Beijing Tiantan Hospital Capital Medical University Beijing China

**Keywords:** alternative splicing, lower‐grade diffuse gliomas, molecular subtype, prognosis

## Abstract

Alternative splicing (AS) is assumed to play important roles in the progression and prognosis of cancer. Currently, the comprehensive analysis and clinical relevance of AS in lower‐grade diffuse gliomas have not been systematically addressed. Here, we gathered alternative splicing data of lower‐grade diffuse gliomas from SpliceSeq. Based on the Percent Spliced In (PSI) values of 515 lower‐grade diffuse glioma patients from the Cancer Genome Atlas (TCGA), we performed subtype‐differential AS analysis and consensus clustering to determine robust clusters of patients. A total of 48 050 AS events in 10 787 genes in lower‐grade diffuse gliomas were profiled. Subtype‐differential splicing analysis and functional annotation revealed that spliced genes were significantly enriched in numerous cancer‐related biological phenotypes and signalling pathways. Consensus clustering using AS events identified three robust clusters of patients with distinguished pathological and prognostic features. Moreover, each cluster was also associated with distinct genomic alterations. Finally, we developed and validated an AS‐related signature with Cox proportional hazards model. The signature, significantly associated with clinical and molecular features, could serve as an independent prognostic factor for lower‐grade diffuse gliomas. Thus, our results indicated that AS events could discriminate molecular subtypes and have prognostic impact in lower‐grade diffuse gliomas.

## 1 INTRODUCTION

Gliomas are the most common and lethal form of malignant primary brain tumours. Despite aggressive therapies with surgical resection, radio and chemotherapy, the treatment outcome is still not satisfactory.^1^, ^2^ Glioblastoma multiforme (GBM), the highly aggressive tumour, accounts for 55% of gliomas with a dismal median survival of 14‐16 months.^3^, ^4^ Due to the diffuse and infiltrative inherence, GBM shows strong resistance to treatment and inevitable recurrence.^5^, ^6^ The 2016 World Health Organization classification of central nervous system tumours uses molecular parameters to define diffuse glioma in addition to histology. Diffuse gliomas are divided into 5 categories: diffuse astrocytoma, *IDH*‐mut; oligodendroglioma, *IDH*‐mut and 1p/19q codeleted; diffuse astrocytoma, *IDH*‐wt; GBM, *IDH*‐mut; and GBM, *IDH*‐wt.^7^ Although increasing studies have found several genetic mutations and deregulated signalling pathways affecting the malignant phenotype of gliomas, the current findings are still insufficient to reveal the molecular basis of gliomagenesis.^8^ To further uncover the transcriptional dissimilarity among subgroups, we undertook an analysis of alternative splicing (AS) in lower‐grade diffuse gliomas.

Alternative splicing (AS) is a post‐transcriptional modification generating multiple mRNA and protein isoforms from a single gene. The vast majority of human genes (approximately 95%) are alternatively spliced.^9^, ^10^ Growing evidence has shown that AS is closely associated with tumorigenesis and progression, especially growth, invasion and metastasis of tumour cells.^11^, ^12^, ^13^ Moreover, AS can serve as valuable biomarkers for cancer classification, diagnosis and prognosis evaluation.^14^, ^15^, ^16^ TCGA SpliceSeq is a web‐based resource that provides an overview of alternative splicing form 33 different tumour types, including available adjacent normal samples. This data set contains seven AS patterns, namely, alternate acceptor site (AA), alternate donor site (AD), alternate promoter (AP), alternate terminator (AT), exon skip (ES), mutually exclusive exons (ME) and retained intron (RI).^17^ Percent Spliced In (PSI) value, a common and intuitive ratio, was introduced to quantify splicing events.^10^

In gliomas, the role of AS remains largely unexplored. Previous studies have mainly focused on alternative spliced isoforms of individual genes. For instance, β splicing of human telomerase reverse transcriptase (*hTERT*) was correlated with clinical parameters and could serve as a prognostic maker in gliomas.^18^ *BAF45d* splicing mediated by polyprimidine tract‐binding protein 1 (*PTBP1*) maintained the undifferentiated cellular state in glioblastoma.^19^ Lineage‐specific *ANXA7* splicing in a constituent of an oncogenic pathway eliminated tumour suppressor functions and promoted glioblastoma progression.^20^ However, comprehensive alterations of AS and their clinical implications in lower‐grade diffuse gliomas have yet to be elucidated.

In this study, we undertook a comprehensive investigation of alternative splicing across lower‐grade diffuse glioma subgroups in the TCGA cohort (n = 515). Using data from SpliceSeq, we identified subtype‐differential AS events and genes involved in different biological functions. Furthermore, we identified three clusters of cases with significant difference in prognosis based on the consensus clustering of AS events. We also developed a 17‐AS event signature for prognostic evaluation using differential AS events associated with overall survival (OS). Our results further highlight the transcriptional difference among subtypes and provide additional biomarker for subtype assignment in lower‐grade diffuse gliomas.

## 2 MATERIALS AND METHODS

### 2.1 Patients and tissues

515 lower‐grade diffuse glioma samples from the TCGA database were included in this study. All these tissue samples and clinicopathologic information were collected with informed consent. The study was conducted in accordance with the Declaration of Helsinki. Study protocols were approved by the ethics committees of the participating institutions.

### 2.2 Data obtaining and processing

The RNA sequencing data, somatic mutation and copy number alterations (CNAs) data, and corresponding clinical information of all collected patients, such as age, gender, histology, isocitrate dehydrogenase (*IDH*) mutation status, 1p/19q status, methylguanine methyltransferase (*MGMT*) promoter status, *TERT* promoter status and survival information, were downloaded from TCGA database (http://cancergemome.nih.gov/). Patient characteristics were summarized in Table S1. The corresponding alternative splicing data were obtained from the SpliceSeq database (http://bioinformatics.mdanderson.org/TCGA SpliceSeq).^10^ The primary PSI data with no more than 25% missing values were analysed, and the missing values were filled up with average PSI value of each event. The samples were then randomly divided into two parts (Table S1), namely training and validation groups, to performing further analyses. All data supporting this study were openly available from TCGA database and SpliceSeq database.

### 2.3 Consensus clustering

Using R package ‘ConsensusClusterPlus’, consensus clustering was performed for class discovery based on the comparison of alternative splicing profile.^21^, ^22^, ^23^ Measured by median absolute deviation (MAD > 0.16), the most variable AS events were retained for subsequent clustering. PSI of each AS event was median‐centred and the similarity between AS profile was quantified using Pearson correlation. The cumulative distribution function (CDF) was constructed for a range from 2 to 10 consensus clusters. The optimal number of clusters was evaluated using CDF and consensus matrices.

### 2.4 AS‐related signature identification

Univariate Cox regression analysis was performed to identify prognosis‐associated AS events (*P* < .05). Then, the Cox proportional hazards model was applied for selecting optimal prognostic AS event set using R package ‘glmnet’, which was suitable for regression analysis of high‐dimensional data.^24^, ^25^, ^26^ After that, risk score of each patient was calculated with the linear combination of the PSI value weighted by their regression coefficients (Coeffs). Risk score = (PSI_AS1_ × Coeff_AS1_) + (PSI_AS2_ × Coeff_AS2_) + … + (PSI_ASn_ × Coeff_ASn_).

### 2.5 Bioinformatic analysis

Gene ontology (GO) analysis was applied for the functional annotation of differential spliced genes among subgroups. Kyoto encyclopedia of genes and genomes (KEGG) analysis was performed to analyse the pathway enrichment (http://david.ncifcrf.gov/).^27^ A *t* test with *P*‐values adjusted by Benjamini‐Hochberg method was employed to identify differential AS events based on the threshold of foldchange more than 2 and adjusted *P*‐value <.05. ROC curve analysis was used to predict OS with R package ‘pROC’. Gene set enrichment analysis (GSEA) was performed to identify gene sets of statistical difference between two groups by using GSEA v3 software (http://www.broadinstitute.org/gsea/index.jsp).^28^ GISTIC2.0 analysis was adopted to assess CNAs associated with clusters. Locus with GISTIC value more than 1 or less than −1 was defined as an amplification or deletion, respectively.^29^

### 2.6 Statistical analysis

Univariate and multivariate Cox regression analyses were conducted to identify independent prognostic factors. Chi‐square test was performed to detect the difference of pathological features between groups. Kaplan‐Meier with 2‐sided log‐rank test was used to assess the OS difference between groups. Cases with missing information were excluded from the corresponding analysis. All statistical analyses were conducted using SPSS, GraphPad Prism 6.0, and R software. *P* < .05 was considered statistically significant.

## 3 RESULTS

### 3.1 Overview of AS events in lower‐grade diffuse gliomas of TCGA cohort

**Figure 1:**
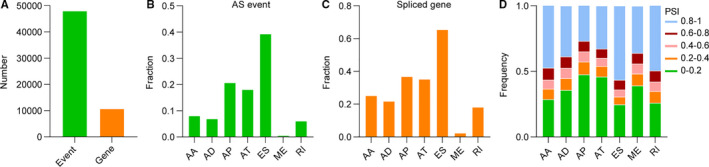
Overview of AS events in lower‐grade diffuse gliomas of TCGA cohort. A, Total number of AS events and spliced genes identified in lower‐grade diffuse gliomas. B and C, Bars represent the fraction of different types of AS events and spliced genes. D, Bars indicate the fraction of AS events of distinct PSI levels. AA, alternate acceptor site; AD, alternate donor site; AP, alternate promoter; AT, alternate terminator; ES, exon skip; ME, mutually exclusive exons; RI, retained intron

To uncover the alternative splicing features of lower‐grade diffuse gliomas, we extensively analysed the AS events data obtained from SpliceSeq database. A total of 48 050 AS events in 10 787 genes were detected (Figure 1A): in detail, 3876 AAs (8%) in 2719 genes, 3351 ADs (7%) in 2353 genes, 9964 APs (21%) in 3976 genes, 8718 ATs (18%) in 3809 genes, 18 931 ESs (39%) in 7073 genes, 273 MEs (1%) in 261 genes and 2937 RIs (6%) in 1970 genes (Figure 1B,C). Interestingly, ES was the most frequently observed AS events, followed by AP and AT events. While ME events were fewest, with only 273 events. In addition, we analysed the detection frequency of AS events with varying PSI levels in all samples. The results showed that events with PSI levels (0‐0.2 and 0.8‐1) constituted the majority of all types of AS events (Figure 1D). Although the number of detected AS events varied in all samples, the pattern of AS types with distinct PSI levels was similar.

### 3.2 The landscape of differentially expressed AS events between subtypes in lower‐grade diffuse gliomas

**Figure 2:**
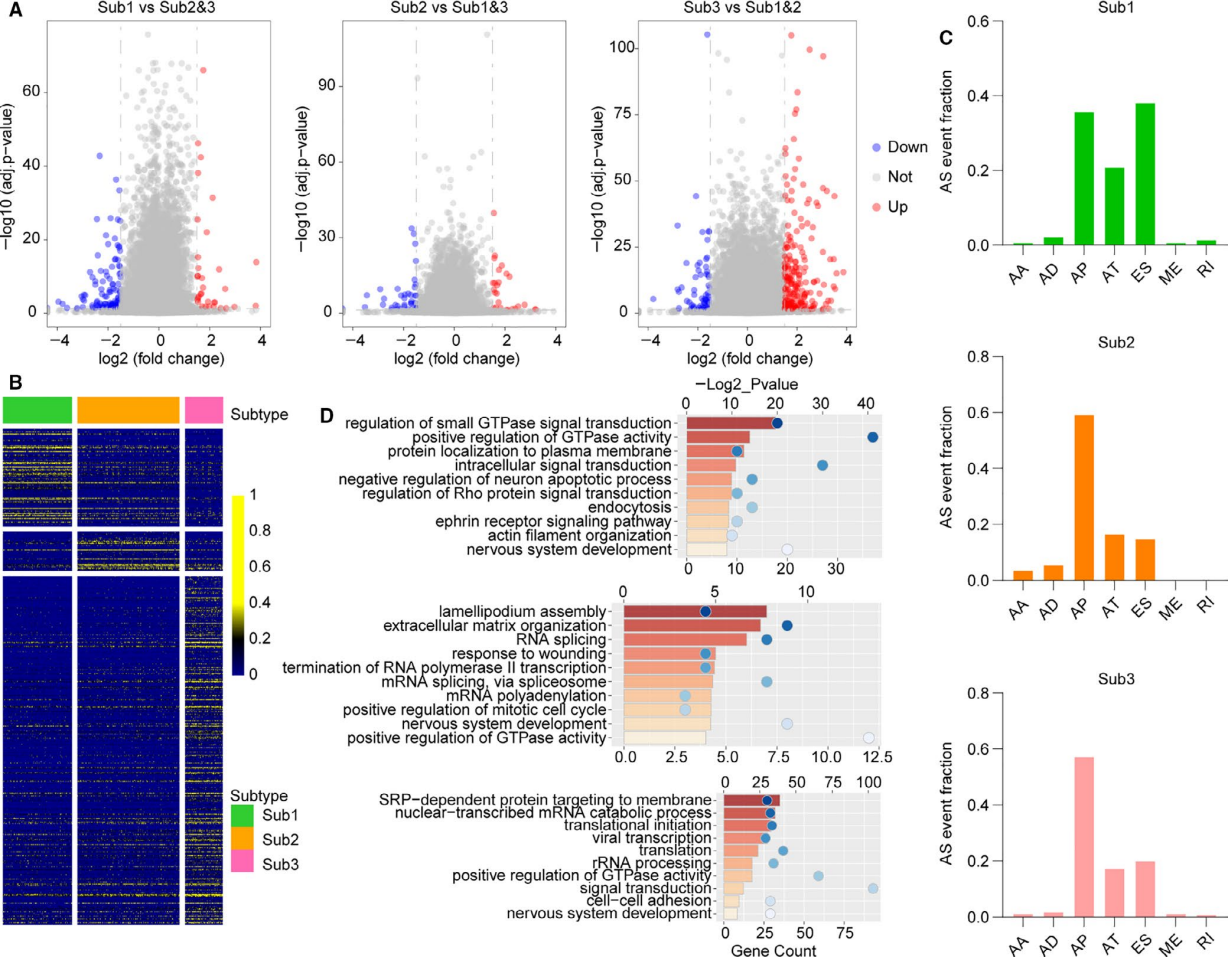
The landscape of differentially expressed AS events in lower‐grade diffuse gliomas. A, Volcano plots represent the differentially spliced AS events of each subtype. Sub1 stands for cases with *IDH* mutation and 1p/19q codeletion, Sub2 for cases with *IDH* mutation and 1p/19q non‐codeletion, Sub3 for *IDH* wild type. B, Heatmap shows the differential AS events identified by comparative analysis (*t* test, Benjamini‐Hochberg method). C, Differentially spliced events in each subtype, bars indicate the proportion of each AS event type. D, GO analyses of genes with differential AS events for each subtype

To portray the full landscape of aberrant AS in lower‐grade diffuse gliomas, we next identified differential AS events across molecular subtypes. We used *t* test (*P*‐values adjusted by Benjamini‐Hochberg method) to compare the PSI value distributions within the 3 molecular groups (foldchange > 2, *P*‐value < .05). As shown in Figure 2A, volcano plots represented the differentially spliced AS events of each subtype. The differential AS events identified by comparative analysis were comprehensively displayed in heatmap using hierarchical clustering (Figure 2B and Table S2). Specifically, 134 differential AS events involved in 107 genes were detected in cases of subtype 1 (*IDH*‐mut and 1p/19q codeleted), 54 differential AS events were observed in subtype 2 (*IDH*‐mut and 1p/19q non‐codeleted), and 478 differential AS events occurred in subtype 3 (*IDH*‐wt). The proportional composition of differential AS types is similar, and AP was the most frequently observed differential AS type (Figure 2C). In addition, some differential AS events within subtypes could be identified in the same genes (for example, *IL11RA* and *KCNIP4*). The Venn diagram and heatmap showed distribution of genes of differential AS events (Figure S1A,B). Single type of differential AS events was observed in most of genes, whereas some genes harboured distinct types of differential AS events (*EEF1D*, *MRPL55*, *SLC14A2*, *ZSCAN18*, *AKAP7*, *ERBB2*, Figure S1C). Some genes were also reported in previous studies in gliomas, such as *SGK1*, *TCF4* and *FGFR1*.^30^, ^31^, ^32^ These results indicated that differential AS events varied in genes and patient groups in lower‐grade diffuse gliomas.

### 3.3 Functional enrichment of differential AS events

To further define the biological functions potentially affected by AS in lower‐grade diffuse gliomas, we performed GO analysis based on genes with differential AS events (foldchange > 1.5, *P*‐value < .05). In patients of subtype 1, we identified a high incidence of GTPase‐mediated signal transduction process affected by AS. Of the top ten statistically significant enrichments, 30% (3/10) were involved in GTPase signal transduction function (Figure 2D). Genes with differential AS events in subtype 2 were mainly enriched in mRNA splicing processing. Instead, genes in subtype 3 patients were annotated to translation and signal transduction (Figure 2D). In addition, nervous system development and regulation of GTPase activity were observed in all groups of patients.

We further performed KEGG analysis to identify pathways in which genes of differential AS events might be involved. Pathways such as Rap1 signalling pathway,^33^, ^34^ pathway in cancer and insulin signalling pathway^35^, ^36^ which were previously implicated in gliomas were significantly enriched in differentially spliced genes in subtype 1 (Figure S2A). Similarly, AS events in subtype 3 comprised crucial pathways reported in gliomas, including Ras signalling pathway^37^, ^38^ and cAMP signalling pathway^39^, ^40^ (Figure S2B). Our analyses revealed that differential AS events participated in many important biological process and pathways involved in glioma pathogenesis.

### 3.4 Consensus clustering of AS events identifies three distinct tumour subgroups

**Figure 3:**
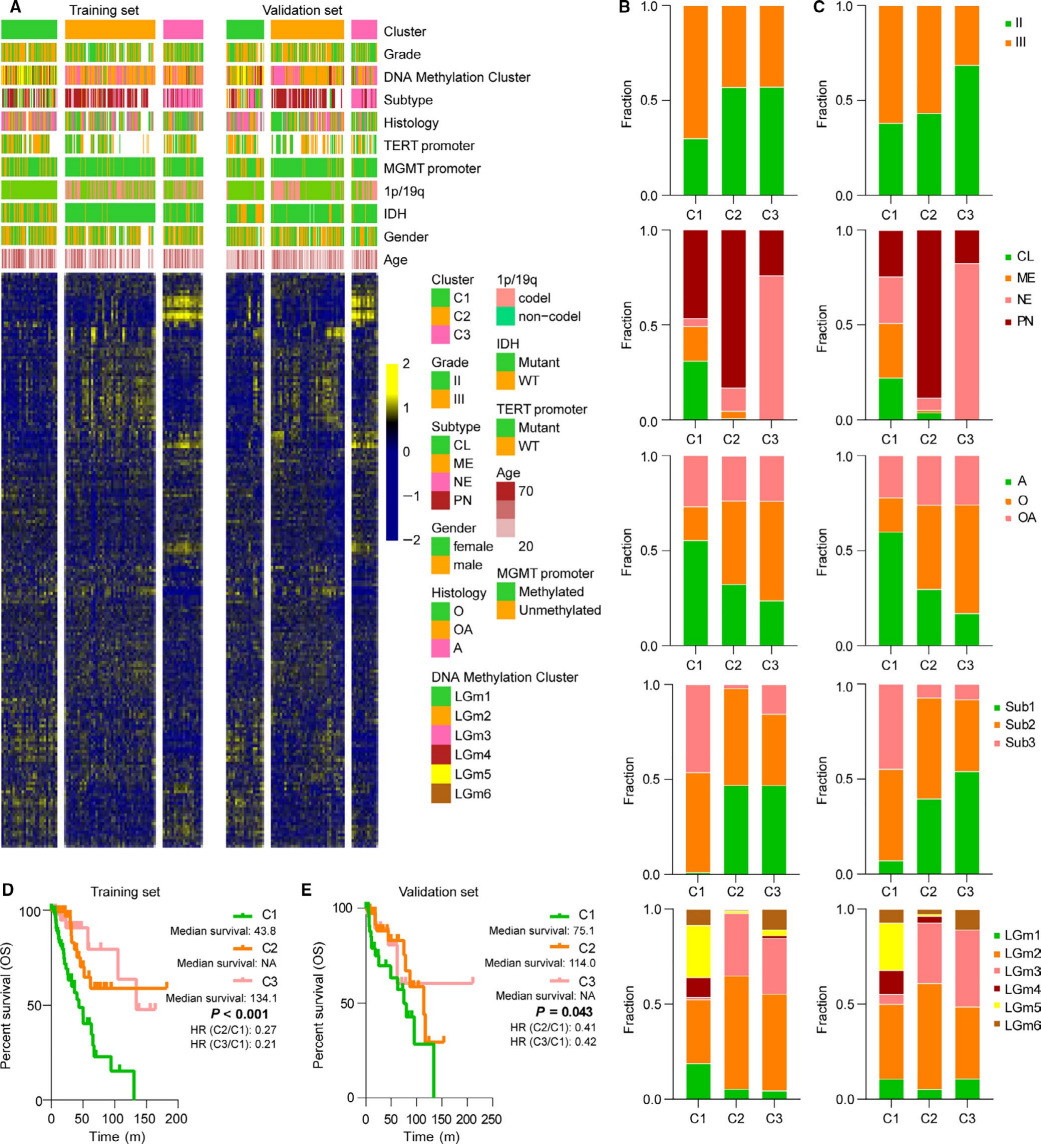
Consensus clustering of AS events identifies three clusters. A, Heatmap of three clusters defined by 4000 AS events (MAD > 0.16). AS order of training set was applied in validation set. B, Bars indicate the fraction of pathological features of different clusters in training set. C, Bars indicate the fraction of pathological features of different clusters in validation set. Sub1 stands for cases with *IDH* mutation and 1p/19q codeletion, Sub2 for cases with *IDH* mutation and 1p/19q non‐codeletion, Sub3 for *IDH* wild type. A stands for astrocytoma; O for oligodendroglioma; AO for oligoastrocytoma. D, Kaplan‐Meier analysis shows distinct overall survival among three clusters in training set. E, Kaplan‐Meier analysis shows distinct overall survival among three clusters in validation set. NA means not reached. HR, hazard ratio. *P*‐value was calculated by the log‐rank test

Considering the significant variation of AS events in lower‐grade diffuse gliomas, we further performed tumour classification based on PSI values through consensus clustering. Tumour samples were randomly distributed into two parts, namely training and validation set. 4000 AS events with highly variable PSI values across samples (MAD > 0.16) were used for subsequent clustering. Among them, AP and ES were the most frequently observed AS events (27.5% and 45.4%, respectively) (Figure S3). Assessing by CDF and consensus matrices (Figure S4), we identified three robust clusters (C1, C2 and C3) produced by AS events data in training set (Figure 3A), because the shape of the CDF curves did not change much beyond this number. The cluster membership of these three groups was associated with distinct prognostic and pathological features (Figure 3B,D). C1, with significantly poor outcome, contained the majority of tumours with classical or mesenchymal subtype. 77% (44/57) *IDH* wild‐type tumours (subtype 3) were enriched in this cluster. In contrast, C2 and C3 showed better outcome and contained more tumours with *IDH* mutation and 1p/19q codeletion (subtype 1). Within them, C2 was mainly comprised of proneural tumours, whereas neural tumours were enriched in C3 cluster. When it came to the classification reported by Ceccarelli et al,^8^ tumours of LGm5 and LGm4 were enriched in C1 cluster, whereas LGm3 was prevalent in C2 and C3 clusters.

To validate our findings in training set, we evaluated the reproducibility of AS‐related clusters in validation set. Applying the same AS event ordering from the training set in the validation set clearly recapitulated the clusters identified in the training set (Figure 3A). Moreover, the obtained clusters displayed similar pattern of pathological and prognostic features with training set (Figure 3C,E). These results suggested that AS events could serve as valuable biomarkers for classification of lower‐grade diffuse gliomas.

### 3.5 Genomic alterations of AS‐related clusters

**Figure 4:**
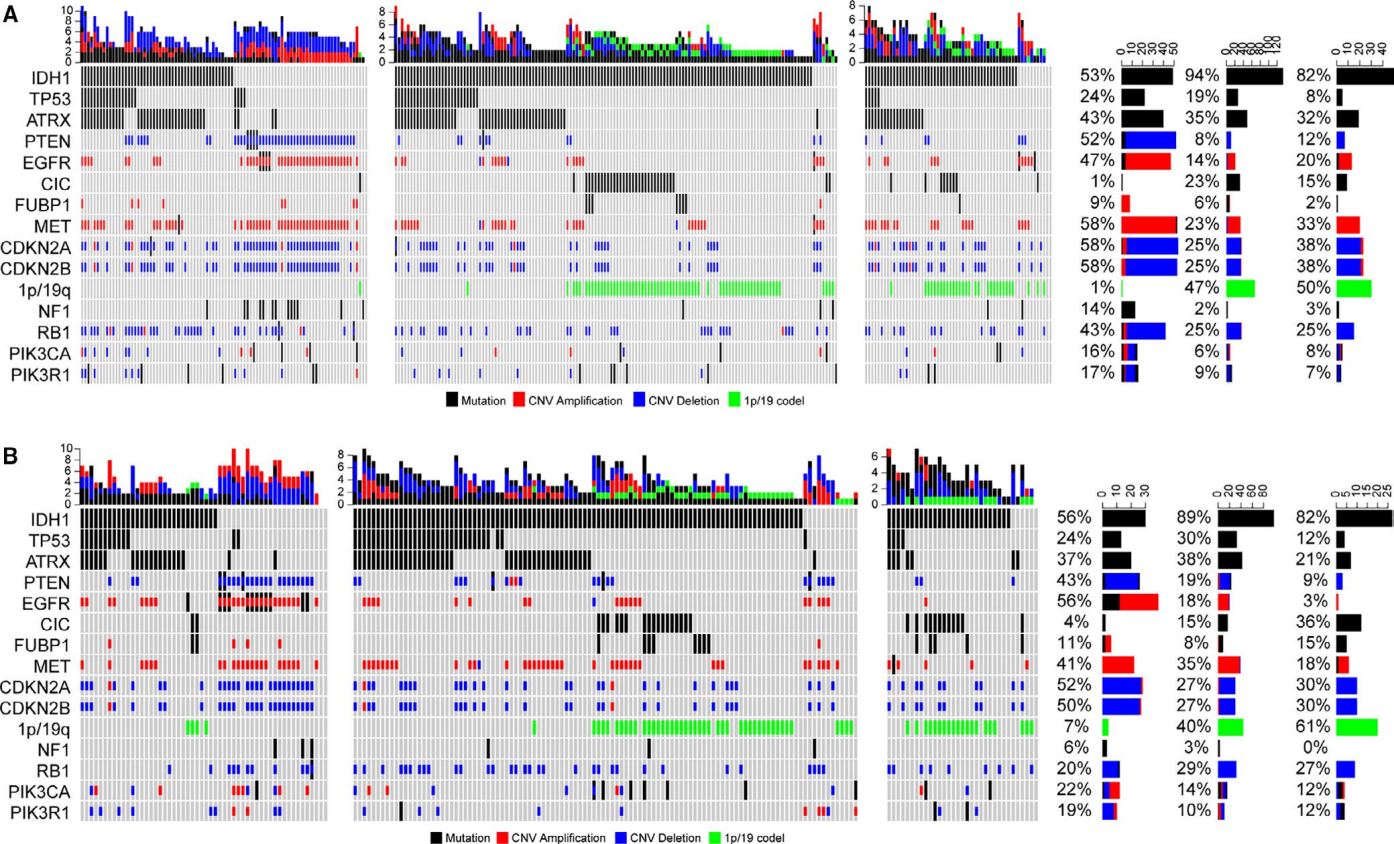
Distinct genomic patterns associate with AS clusters. A, Differential somatic mutations and copy number variations analyses within three AS clusters of Training set (Fisher test). B, Differential somatic mutations and copy number variations analyses within three AS clusters of validation set (Fisher test)

To explore the association between AS‐related cluster and genomic alterations, we analysed the somatic mutations and copy number variations (CNVs) data form TCGA database. First, we compared the frequency of mutations across three AS clusters of training set. Mutations in *NF1*, *EGFR*, *TP53* and *PTEN* occurred more frequently in C1. In contrast, *IDH*, *CIC*, *FUBP1* mutation and 1p/19q codeletion were significantly enriched in C2 and C3 clusters (Figure 4A). Additionally, we observed higher frequency of *TP53* mutation in C2 compared with C3 cluster. Moreover, CNV analysis revealed distinct chromosomal alteration patterns among three clusters. As shown in Figure 4A, C1 showed more frequently deleted or amplified genes, such as *PTEN*, *EGFR*, *MET*, *CDKN2A*, *CDKNA2B*, *RB1*, *PIK3CA* and *PIK3R1*. We next sought to dissect the genomic alterations of each cluster in validation set and obtained consistent results (Figure 4B). These findings confirmed the association between AS‐related cluster and genomic alterations.

### 3.6 Identification of an AS‐related prognostic signature in lower‐grade diffuse gliomas

**Figure 5:**
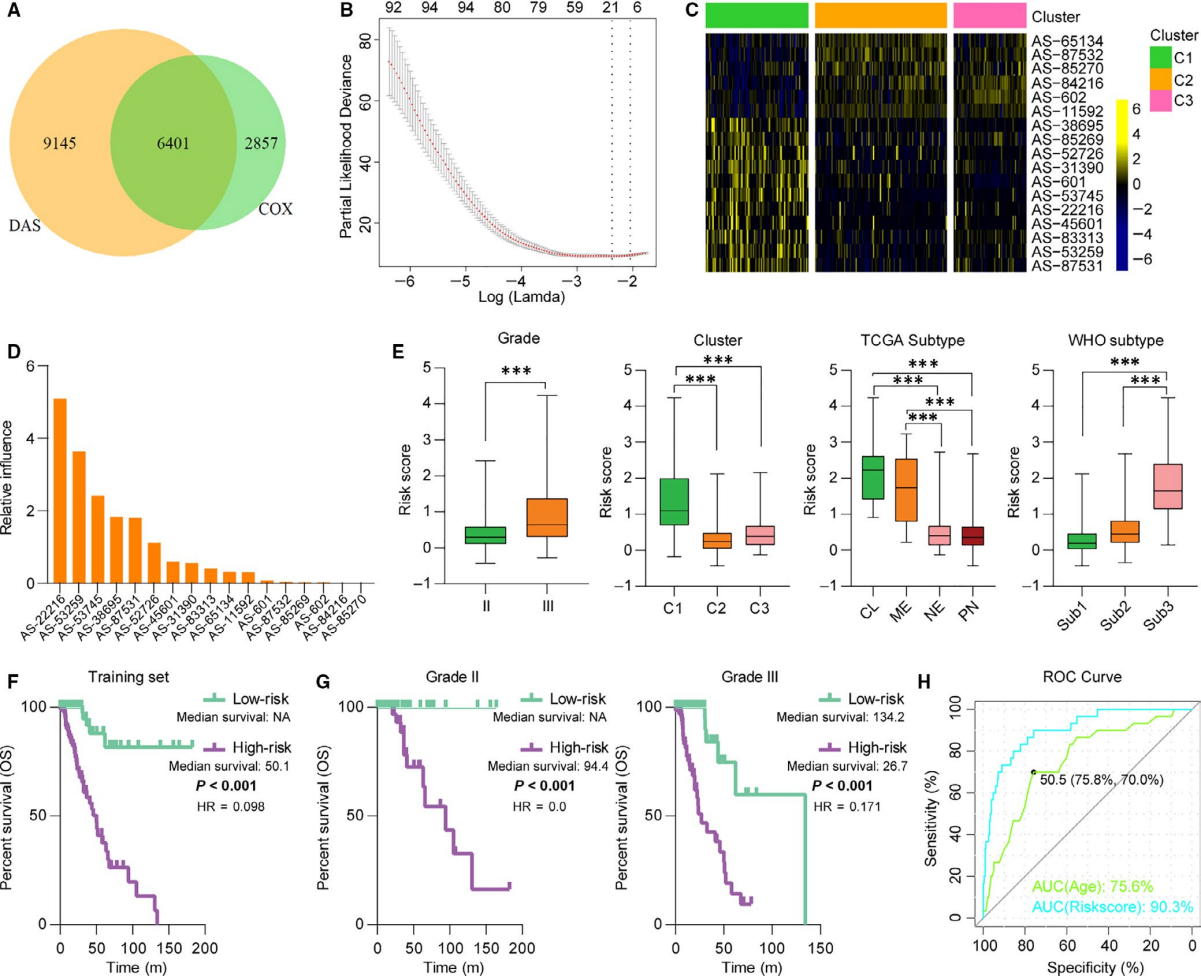
Identification of an AS‐related signature by Cox proportional hazards model in training set. A, Venn diagraph shows 6401 differential AS events significantly correlated with patients’ overall survival in univariate Cox regression analysis. B, Cross‐validation for tuning parameter selection in the proportional hazards model. C, Heatmap exhibits the 17 AS events of the identified signature. D, Relative influence of each AS events of the identified signature. E, Distribution of risk scores in cases stratified by AS cluster, grade, TCGA and WHO subtype. CL, classical; ME, mesenchymal; NE, neural; PN, proneural; Sub1, *IDH* mutant and 1p/19q codeleted; Sub2, *IDH* mutant and 1p/19q non codeleted; Sub3, *IDH* wild type. *P < .05; **P < .01; ***P < .001. F and G, Survival analysis of the signature in diffuse LGG and tumours stratified by grade. HR, hazard ratio. *P*‐value was calculated by the log‐rank test. H, ROC curve analysis of age and risk score. AUC, area under the curve

Considering the strong link between patients’ prognosis and AS events, we proposed to construct an AS‐related signature for outcome assessment. First, we determined the clinical significance of AS events in lower‐grade diffuse gliomas. Consequently, 9258 AS events were significantly correlated with patients’ survival in univariate Cox regression analysis (*P* < .05), wherein 3983 AS events were favourable (hazard ratio < 1) and 5275 AS events were unfavourable (Table S3). *t* Test (Benjamini‐Hochberg method) identified 15 546 differential AS events between C1 and C2/C3, wherein 6401 AS events were associated with patients’ outcome (Figure 5A). Additionally, ES events were major components of these AS events correlated with OS, and most of these events showed a range of PSI level (0‐0.2 and 0.8‐1) (Figure S5). We next applied a Cox proportional hazards model for selecting AS events with best prognostic value (Figure 5B). Consequently, a 17‐AS event signature was identified and the risk score was calculated with their PSI values and regression coefficients (Figure 5C and Table 1). The relative influence of each AS event was showed in terms of the absolute value of coefficients (Figure 5D). Multivariate Cox regression analyses found that most of these 17 AS events were independent factors of prognosis (Table S4). High scores were enriched in tumours of grade III, C1, classical and mesenchymal or *IDH* wild type (Figure 5E). Kaplan‐Meier analysis showed that high scores implied significantly poorer outcome in patients of lower‐grade diffuse gliomas or stratified tumours (Figure 5F,G and Figure S6). In addition, the acquired signature had higher AUC compared with other factors (age) (Figure 5H). Multivariate Cox regression analysis also confirmed the independent prognostic value of this AS signature (Table 2). We further applied this signature into validation set and found consistent results (Figure S7 and Table 2). These data demonstrated the superior performance of AS signature for prognosis prediction, highlighting the importance of AS event in determining survival.

**Table 1:** 17 AS events of the signature identified by Cox proportional hazards model

AS ID	Splice type	Gene symbol	Exons	Coefficient
601	AT	KIF1B	25	0.075764381
602	AT	KIF1B	52.2	−0.027303763
11 592	ES	PCDH15	41:44:00	−0.308824544
22 216	ES	ITGA7	29	5.09422293
31 390	AP	KIF23	1	0.5576822
38 695	ES	RPAIN	5	1.835337004
45 601	AP	TCF4	15	0.597675559
52 726	ES	FAM49A	3	1.11645802
53 259	AT	HNRNPLL	6.3	3.627912308
53 745	AP	UGP2	2	2.420392983
65 134	ME	PCBP4	3|4.1:4.2	−0.318481153
83 313	AT	NGR1	20	0.411772394
84 216	AD	TCEB1	1.2	−0.002534794
85 269	AP	KHDRBS3	1	0.027571605
85 270	AP	KHDRBS3	2	−0.001059352
87 531	AT	PSMB7	7.2	1.797911175
87 532	AT	PSMB7	8	−0.036105776

**Table 2:** Univariate and multivariate Cox regression analysis of clinical pathologic features for OS in lower‐grade diffuse gliomas

Characteristics	Training set						Validation set					
	Univariate analysis			Multivariate analysis			Univariate analysis			Multivariate analysis		
	HR	95% CI	*P*‐value	HR	95% CI	*P*‐value	HR	95% CI	*P*‐value	HR	95% CI	*P*‐value
Age	1.063	1.038‐1.088	**<.001**	1.049	1.015‐1.084	**.004**	1.065	1.036‐1.095	**<.001**	1.095	1.045‐1.147	**<.001**
Gender	1.011	0.568‐1.801	.97				0.838	0.413‐1.699	.623			
*MGMT* promoter	0.281	0.147‐0.536	**<.001**	1.226	0.529‐2.839	.635	0.803	0.334‐1.931	.625			
Grade	0.192	0.096‐0.385	**<.001**	0.400	0.167‐0.958	**.040**	0.563	0.269‐1.180	.128			
*IDH*	0.066	0.031‐0.139	**<.001**	0.835	0.221‐3.162	.791	0.253	0.118‐0.540	**<.001**	1.643	0.225‐12.006	.625
1p/19q	0.288	0.127‐0.652	**.003**	0.464	0.153‐1.406	.174	0.759	0.334‐1.728	.511			
*TERT* promoter	1.247	0.649‐2.398	.507				2.584	1.030‐6.484	**.043**	0.851	0.296‐2.448	.765
Cluster			**<.001**			**.046**			**.053**			.449
C1 vs C3	5.367	2.138‐13.471	**<.001**	2.402	0.786‐7.338	.124	2.396	0.792‐7.243	.122	0.593	0.093‐3.779	.581
C2 vs C3	1.460	0.523‐4.076	.471	4.501	1.373‐14.749	**.013**	0.992	0.313‐3.142	.989	1.308	0.243‐7.046	.755
Risk score	7.866	5.147‐12.020	**<.001**	5.948	3.332‐10.618	**<.001**	2.494	1.611‐3.859	**<.001**	4.070	1.433‐11.562	**.008**

Notes

Gender: male, female; Grade: II, III; *IDH*: mutant, wild type; *MGMT* promoter: methylated, unmethylated; 1p/19q: codeleted, non‐codeleted; *TERT* promoter: mutant, wild type. Values in bold, statistically significant.

Bold indicates statistical significant value.

## 4 DISCUSSION

Alternative splicing events have previously been shown to contribute to tumorigenesis and progression. Most studies have mainly focused on alternative spliced isoforms of individual genes,^18^, ^19^, ^20^ but only a few comprehensive studies are available.^41^, ^42^ The complete landscape of alternative splicing complexity and its clinical significance are still missing in lower‐grade diffuse gliomas.

In this study, we presented the integrated portrait of alternative splicing in lower‐grade diffuse gliomas using SpliceSeq data. Seven types of AS events, namely alternate acceptor site, alternate donor site, alternate promoter, alternate terminator, exon skip, mutually exclusive exons and retained intron, were analysed, and events with PSI levels (0‐0.2, 0.8‐1) formed the majority of all AS types. We further identified differential AS events across each lower‐grade glioma subgroup based on the distribution of PSI values. Functional enrichment analysis of genes with differential AS events identified numerous cancer‐related biological phenotypes and signalling pathways. In future work, we propose to select some differential splicing events for further expression and functional validation (including knockdown and over‐expression assays), for uncovering the possible roles of these isoforms on glioma progression.

Growing evidence suggests that alternative splicing events could discriminate molecular subtypes in various cancers. Leivonen et al reported that AS can differentiate subtypes in diffuse large B‐cell lymphoma.^14^ In medulloblastoma, unsupervised hierarchical clustering of AS events accurately assigns cases to their correct subgroup.^15^ Additionally, unsupervised clustering using isoform‐level gene expression profiles recaptured molecular subgroups with improved prognostic stratification in glioblastoma multiforme.^43^ Here, lower‐grade diffuse gliomas were subjected to consensus clustering based on the PSI values of AS events. Consequently, three clusters were identified. Survival analysis revealed that cases of these three clusters exhibited significantly different OS. Meanwhile, distinct molecular and clinical characteristic were observed in cases of clusters. These results implied that AS events could serve as a potential biomarker for molecular stratification in lower‐grade diffuse gliomas.

To understand the impact of somatic variants on alternative splicing events, we performed association study of tumour variants with AS variants across the genome. More somatic mutations and CNVs were observed in cases of C1. The oncogenic driver *EGFR* ^44^, ^45^ was detected with high amplification peaks in C1 tumours. Meanwhile, a deletion peak of *CDKN2A* and *CDKN2B* was also observed in these tumours.^46^ Therefore, genomic alterations were significantly associated with AS status, implying an underlying regulatory relationship between them.

Recent studies have discovered that alternative splicing events could be more effective as diagnostic and prognostic markers than corresponding genes. Valuable AS signatures were developed in numerous cancers, including ovarian cancer,^47^ bladder urothelial carcinoma,^16^ GBM^43^ and non‐small cell lung cancer.^48^ Based on the differential AS events, we built a signature that could stratify patients with high or low risk of poor outcome. An elastic net regression Cox model was used to increase the predictive performance of prognostic index,^22^ and the obtained 17 AS events showed a cumulative effect on survival prediction. Subsequent analyses demonstrated that the 17‐AS event signature could serve as a powerful prognostic indicator and stratify patients for AS‐targeted therapies in future.

We further compared gene expression between high‐ and low‐risk cases. Based on the top genes of differential expression (fold change > 1.5, *P* < .05) identified by SAM, GO analysis revealed that cell division, DNA replication and immune response were significantly enriched in high‐risk gliomas, whereas the low‐risk cases showed enrichment of oxidation‐reduction process and chemical synaptic transmission (Figure S8A,B). Meanwhile, GSEA confirmed these findings (Figure S8C,D).

In summary, we systematically characterized the dysregulation of AS events and its biological and clinical significance in lower‐grade diffuse gliomas. Our data demonstrated that differentially expressed AS events could act as risk indicator of patients’ survival and further highlighted the transcriptional diversity within subgroups of gliomas.

## CONFLICT OF INTEREST

The authors declare that they have on conflicts of interest.

## AUTHOR CONTRIBUTIONS

**Zheng Zhao:** Data curation (equal); Formal analysis (equal); Methodology (equal). **Guan‐Zhang Li:** Data curation (equal); Formal analysis (equal); Methodology (equal). **Yu‐Qing Liu:** Data curation (equal); Formal analysis (equal); Methodology (equal). **Ruo‐Yu Huang:** Data curation (equal); Formal analysis (equal); Methodology (equal). **Kuan‐Yu Wang:** Resources (equal); Validation (equal). **Hao‐Yu Jiang:** Resources (equal); Validation (equal). **Ren‐Peng Li:** Resources (equal); Validation (equal). **Rui‐Chao Chai:** Formal analysis (equal); Validation (equal); Writing‐original draft (equal). **Chuan‐Bao Zhang:** Formal analysis (equal); Validation (equal); Writing‐original draft (equal). **Fan Wu:** Conceptualization (lead); Supervision (lead); Writing‐review & editing (equal).

## Supporting information

Supplementary Material

## Data Availability

All data supporting this study were openly available from TCGA database (http://cancergemome.nih.gov/) and SpliceSeq database (http://bioinformatics.mdanderson.org/TCGA SpliceSeq).
