# Supplementary material for: Characterization and prognostic significance of alternative splicing events in lower‐grade diffuse gliomas
Source: J Cell Mol Med. 2020 Oct 2;24(22):13171–80. doi: 10.1111/jcmm.15924 (PMC7701518; doi:10.1111/jcmm.15924)
Supplement: Supplementary file 1 — Supplementary Material [file JCMM-24-13171-s001.docx]

**Supplementary data**

**Supplementary figures**

Figure S1. Genes with differential AS events in subtypes.

(A) Venn diagram shows the distribution of genes with differential AS events. Two Genes (*IL11RA* and *KCNIP4*) harbor differential AS events in all subtypes. (B) Heatmap shows example genes (*IL11RA* and *KCNIP4*) with differential AS events. (C) Heatmap shows example genes with different AS types. Cells in green color represent AS types.


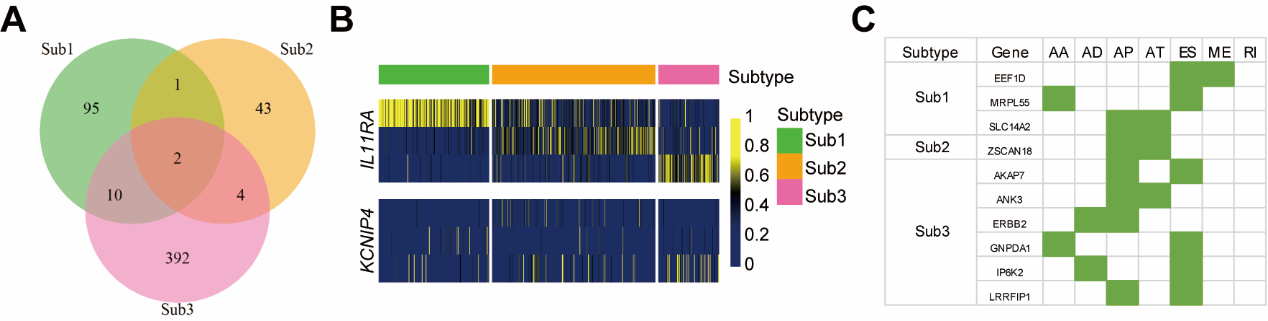


Figure S2. Enrichment analysis of differentially spliced genes in different molecular subtype.

(A and B) KEGG analysis shows the differentially enriched pathways of Sub1 and Sub3. Sub1 stands for cases with *IDH* mutation and 1p/19q codeletion, Sub 3 for *IDH* wide-type.


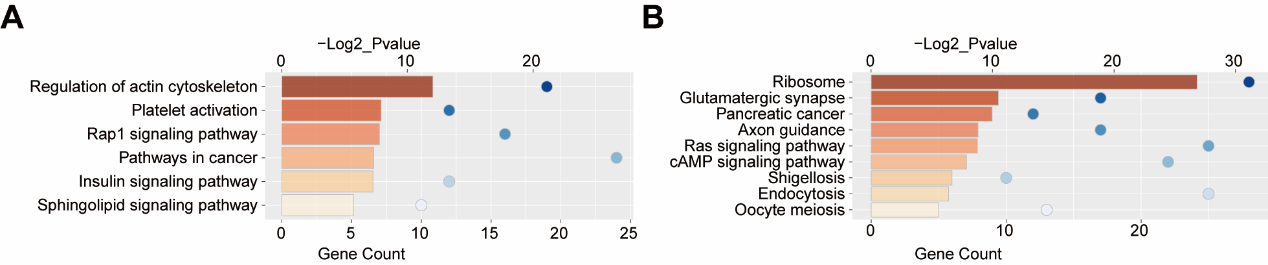


Figure S3. 4000 AS events with highly variable PSI values in training set.

(A) Number of AS events and spliced genes in training set. (B and C) Bars represent the fraction of different types of AS events and spliced genes. (D) Bars indicate the fraction of AS events of distinct PSI levels. AA stands for alternate acceptor site, AD for alternate donor site, AP for alternate promoter, AT for alternate terminator, ES for exon skip, ME for mutually exclusive exons and RI for retained intron.


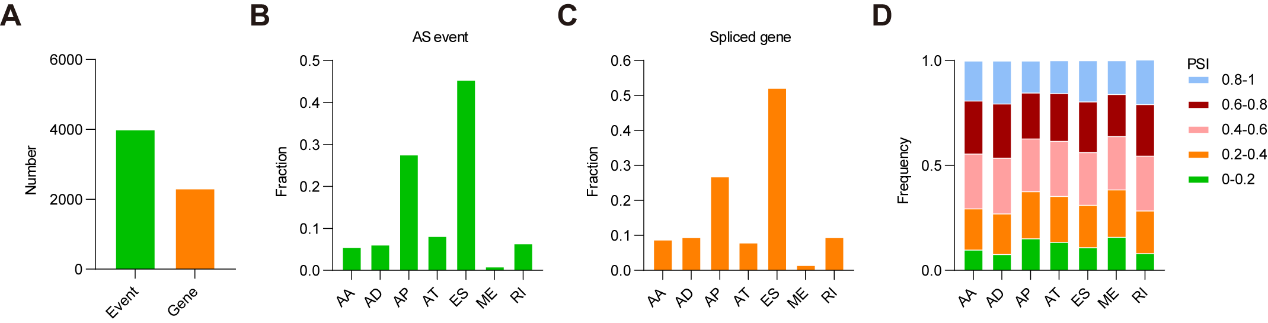


Figure S4. Consensus clustering based on PSI of AS events in training set.

(A) Clustering matrix for k = 2 to k = 10. (B) CDF curve for k = 2 to k = 10. (C) Relative change in area under CDF curve for k = 2 to k = 10. (D) Tracking plot for k = 2 to k = 10.


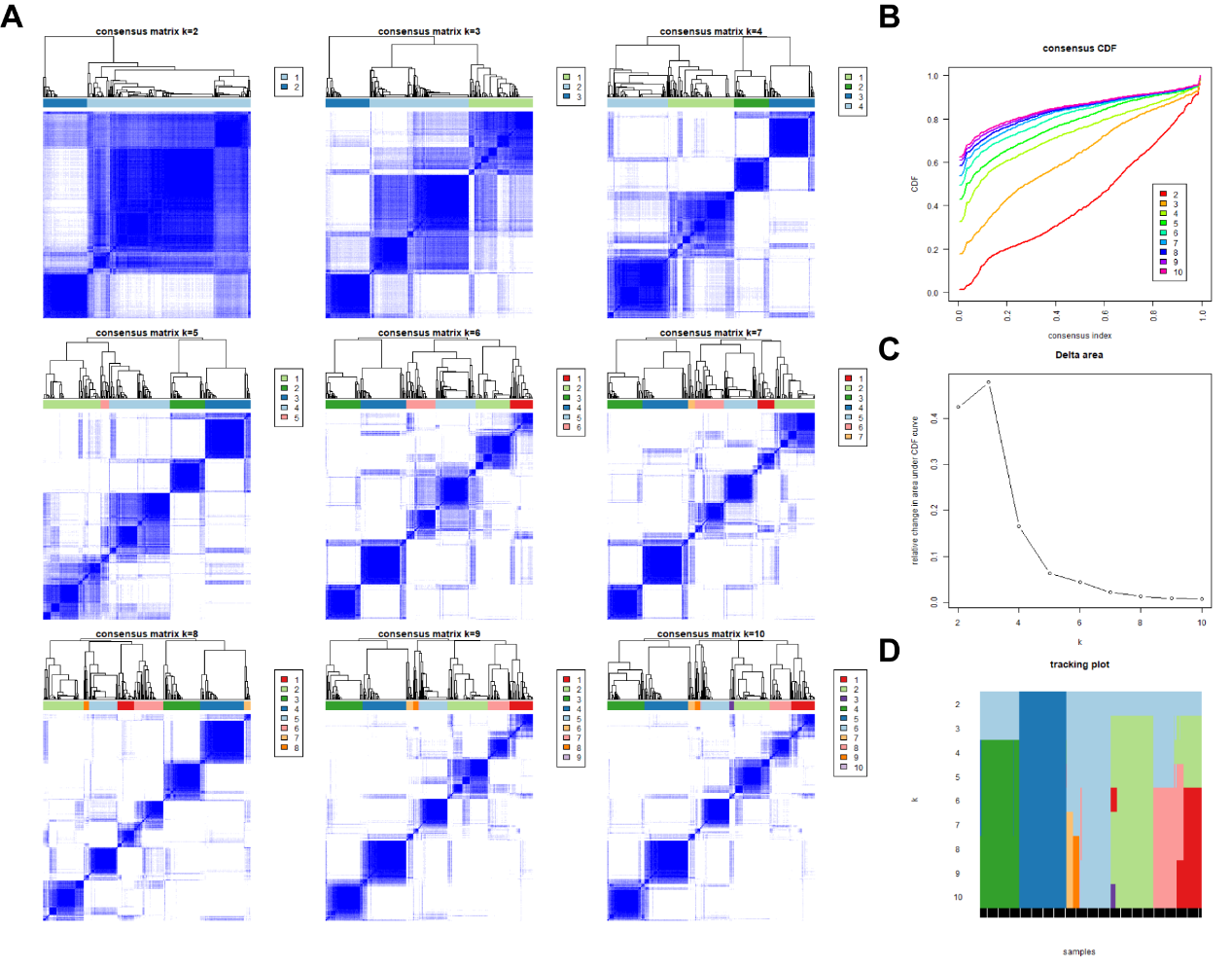


Figure S5. Differential AS events correlated with patients’ overall survival.

(A) Total number of differential AS events and spliced genes correlated with patients’ overall survival identified in training set. (B and C) Bars represent the fraction of different types of AS events and spliced genes correlated with patients’ overall survival. (D) Bars indicate the fraction of AS events of distinct PSI levels. AA stands for alternate acceptor site, AD for alternate donor site, AP for alternate promoter, AT for alternate terminator, ES for exon skip, ME for mutually exclusive exons and RI for retained intron.


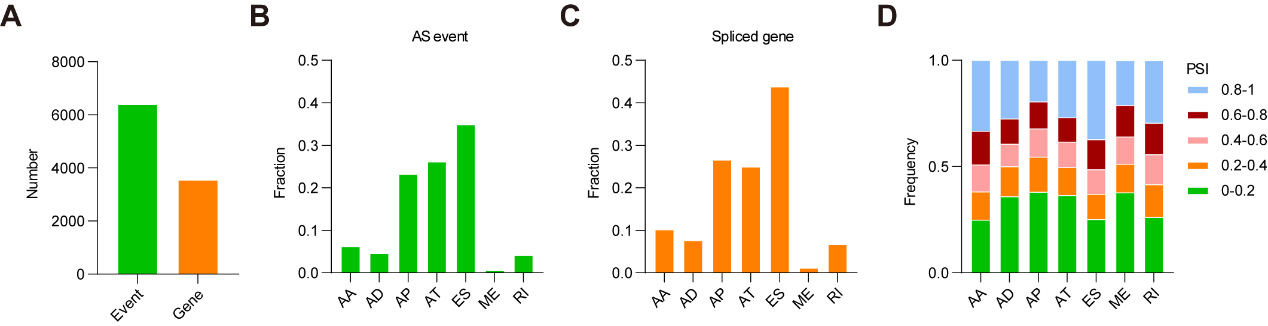


Figure S6. Survival analysis in cases stratified by AS cluster and WHO subtype.

(A and B) Kaplan-Meier analyses of tumors stratified by AS cluster and WHO subtype in training set. (C and D) Kaplan-Meier analyses of tumors stratified by AS cluster and WHO subtype in training set. Sub1 stands for cases with *IDH* mutation and 1p/19q codeletion, Sub2 for cases with *IDH* mutation and 1p/19q non-codeletion, Sub3 for *IDH* wide-type. *P* value was calculated by the log-rank test.


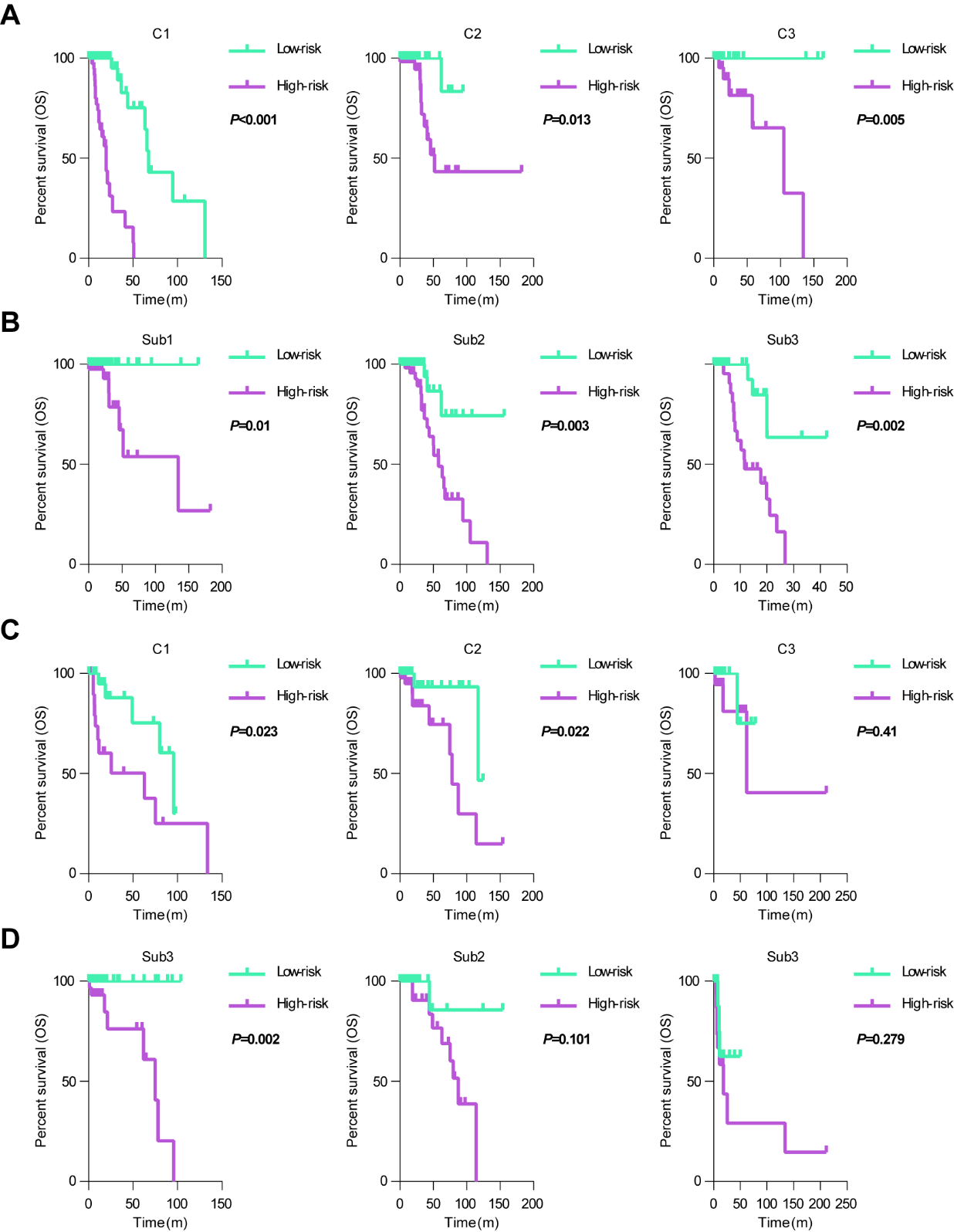


Figure S7. Validation of AS-related signature in validation set.

(A) Heatmap shows 17 AS events of the signature. (B) Distribution of risk scores in patients stratified by AS cluster, grade, TCGA and WHO subtype. ****P* < 0.001. (C and D) Survival analysis of the immune signature in diffuse LGG and tumors stratified by grade. *P* value was calculated by the log-rank test. (E) ROC curve analysis of age and risk score. AUC, area under the curve.


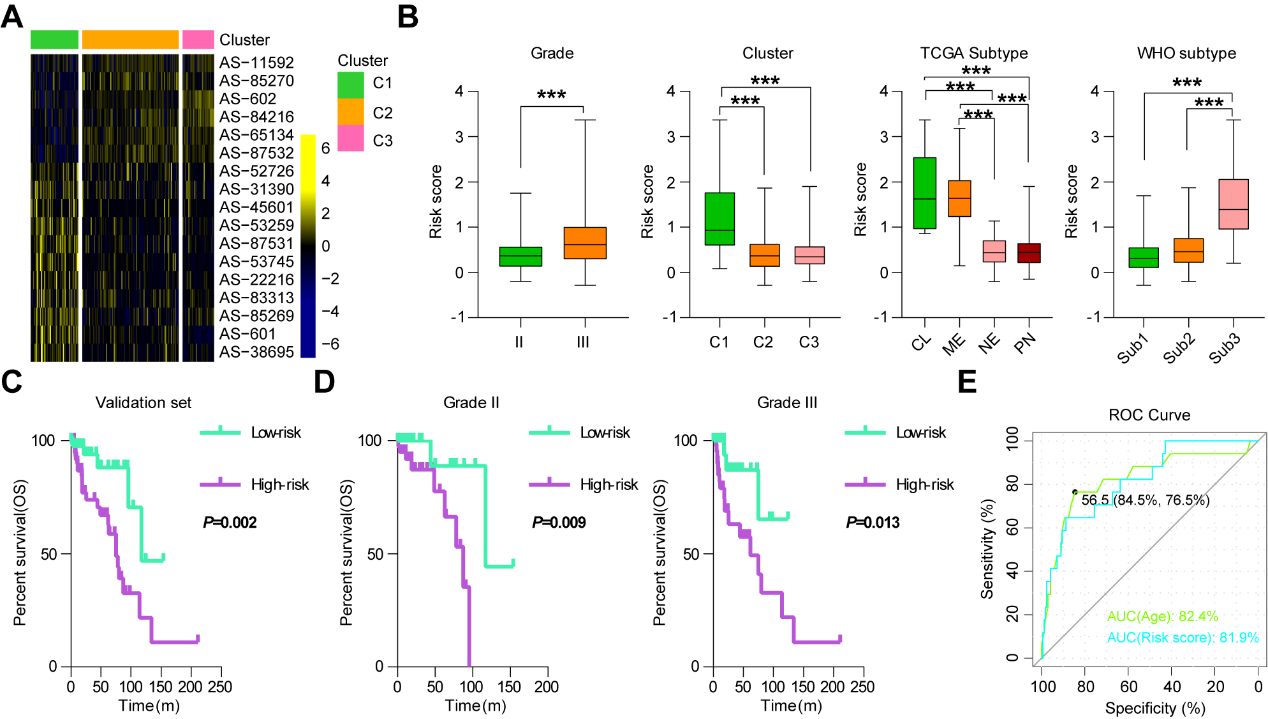


Figure S8. Functional enrichments between high and low-risk cases.

(A and B) Gene ontology (GO) and GSEA analysis of differential genes between high and low-risk cases in Training set. (C and D) Gene ontology (GO) and GSEA analysis of differential genes between high and low-risk cases in validation set.


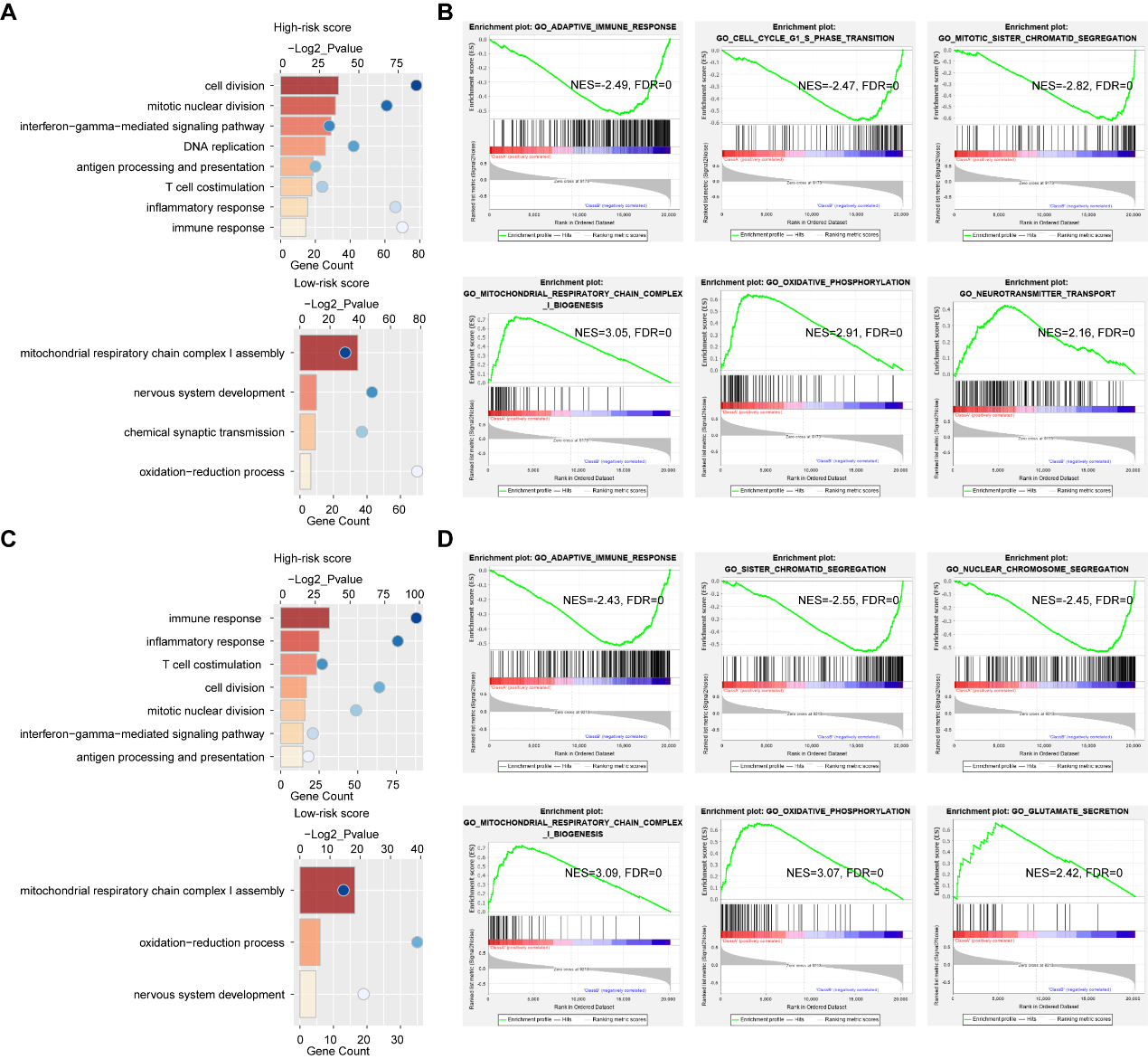


**Supplementary tables**

Table S1. Clinical and molecular characteristics of patients included in this study.

| **Characteristics** | **All (n=515)** | **Training set (n=309)** | **Validation set (n=206)** |
| --- | --- | --- | --- |
| **Age** |  |  |  |
| ≤41 | 237 | 140 | 97 |
| >41 | 219 | 127 | 92 |
| NA | 59 | 42 | 17 |
| **Gender** |  |  |  |
| Male | 256 | 152 | 104 |
| Female | 200 | 115 | 85 |
| NA | 59 | 42 | 17 |
| **TCGA Subtype** |  |  |  |
| Classical | 36 | 23 | 13 |
| Mesenchymal | 31 | 17 | 14 |
| Proneural | 220 | 135 | 85 |
| Neural | 105 | 61 | 44 |
| NA | 123 | 73 | 50 |
| ***IDH* status** |  |  |  |
| Mutant | 419 | 251 | 168 |
| WT | 93 | 57 | 36 |
| NA | 3 | 1 | 2 |
| ***MGMT* promoter status** |  |  |  |
| Methylated | 425 | 256 | 169 |
| Unmethylated | 90 | 53 | 37 |
| **1p/19q status** |  |  |  |
| Non-codeleted | 346 | 208 | 138 |
| Codeleted | 169 | 101 | 68 |
| ***TERT* promoter status** |  |  |  |
| Mutant | 130 | 81 | 49 |
| WT | 162 | 93 | 69 |
| NA | 223 | 135 | 88 |

Table S2. The identified differential alternative splicing events (top 15 ranged by fold change) between subtypes.

| **Sub1 vs Sub 2&3** | | **Sub 2 vs Sub 1&3** | | **Sub 3 vs Sub 1&2** | |
| --- | --- | --- | --- | --- | --- |
| **AS ID** | **FDR** | **AS ID** | **FDR** | **AS ID** | **FDR** |
| 90229 | 0 | 15416 | 0.021 | 89864 | 0 |
| 75993 | 0 | 88767 | 0.021 | 63742 | 0.09 |
| 66436 | 0 | 52400 | 0 | 11842 | 0 |
| 75141 | 0.047 | 3413 | 0 | 81422 | 0 |
| 14546 | 0 | 96827 | 0 | 12691 | 0 |
| 88981 | 0.047 | 10595 | 0 | 60311 | 0 |
| 81837 | 0 | 82643 | 0 | 64918 | 0 |
| 14608 | 0 | 60280 | 0 | 67421 | 0 |
| 10151 | 0 | 90666 | 0 | 88463 | 0 |
| 52668 | 0 | 52402 | 0 | 46924 | 0 |
| 96813 | 0 | 88768 | 0 | 240 | 0 |
| 10140 | 0 | 60787 | 0 | 38960 | 0 |
| 10139 | 0 | 73965 | 0 | 27780 | 0 |
| 69946 | 0.047 | 28637 | 0 | 43726 | 0.019 |
| 10159 | 0 | 60970 | 0 | 83529 | 0.007 |

Table S3. Alternative splicing events significantly (top 15 ranged by HR) correlated with patients’ survival in univariate Cox regression analysis.

| **Favorable AS events** | | **Unfavorable AS events** | |
| --- | --- | --- | --- |
| **AS ID** | ***P*-value** | **AS ID** | ***P*-value** |
| 31281 | 1.05E-05 | 16472 | 0.048675831 |
| 88501 | 6.37E-05 | 13713 | 0.041267496 |
| 46504 | 0.000209003 | 73697 | 0.04165605 |
| 33174 | 0.000929026 | 65684 | 0.049958829 |
| 89831 | 0.00111888 | 61397 | 0.041379108 |
| 27224 | 0.001150619 | 45146 | 0.038732638 |
| 48382 | 0.001152989 | 13614 | 0.035274529 |
| 16336 | 0.001703701 | 87341 | 0.042030565 |
| 87633 | 0.004136115 | 95931 | 0.024439492 |
| 14255 | 0.004772402 | 87343 | 0.033709518 |
| 50953 | 0.005151879 | 52240 | 0.03736345 |
| 36073 | 0.006323734 | 31236 | 0.044590474 |
| 41914 | 0.007439724 | 60971 | 0.046599836 |
| 74084 | 0.008728227 | 49115 | 0.030843938 |
| 75123 | 0.011255684 | 11373 | 0.03034516 |

Table S4. Univariate and multivariate Cox regression analysis of 17 AS events for OS in training set.

| **AS ID** | **Univariate analysis** | | | **Multivariate analysis** | | |
| --- | --- | --- | --- | --- | --- | --- |
|  | **HR** | **95% CI** | ***P*-value** | **HR** | **95% CI** | ***P*-value** |
| 601 | 2.225E7 | 2.131E5-2.322E9 | **<0.001** | 2.249E4 | 110.344-4.855E6 | **<0.001** |
| 602 | 0.000 | 0.000-0.000 | **<0.001** | 0.000 | 0.000-0.009 | **<0.001** |
| 11592 | 0.040 | 0.016-0.100 | **<0.001** | 0.132 | 0.045-0.392 | **<0.001** |
| 22216 | 3.119E6 | 6.615E4-1.471E8 | **<0.001** | 4.510E4 | 140.736-1.445E7 | **<0.001** |
| 31390 | 42.550 | 12.928-140.037 | **<0.001** | 7.292 | 2.069-25.701 | **0.002** |
| 38695 | 5.106E6 | 7.499E4-3.476E8 | **<0.001** | 3.882E4 | 133.215-1.131E7 | **<0.001** |
| 45601 | 3.492E4 | 1.312E3-9.292E5 | **<0.001** | 430.139 | 12.248-1.511E4 | **0.001** |
| 52726 | 1.374E9 | 3.901E5-4.841E12 | **<0.001** | 8.275E3 | 0.887-7.722E7 | 0.053 |
| 53259 | 1.499E21 | 3.259E15-6.898E26 | **<0.001** | 2.432E10 | 1.638E4-3.610E16 | **0.001** |
| 53745 | 7.657E4 | 5.259E3-1.107E6 | **<0.001** | 517.793 | 11.948-2.244E4 | **0.001** |
| 65134 | 0.000 | 0.000-0.001 | **<0.001** | 0.001 | 0.000-0.044 | **0.001** |
| 83313 | 139.532 | 25.413-766.123 | **<0.001** | 19.959 | 3.173-125.558 | **0.001** |
| 84216 | 0.00 | 0.00-0.003 | **<0.001** | 0.009 | 0.000-0.276 | **0.007** |
| 85269 | 1.245E5 | 1.211E3-1.280E7 | **<0.001** | 223.900 | 1.555-3.224E4 | **0.033** |
| 85270 | 0.000 | 0.000-0.001 | **<0.001** | 0.004 | 0.000-0.643 | **0.033** |
| 87531 | 8.936E5 | 2.488E5-3.210E7 | **<0.001** | 2.553E3 | 43.086-1.513E5 | **<0.001** |
| 87532 | 0.000 | 0.000-0.00 | **<0.001** | 0.000 | 0.000-0.023 | **<0.001** |
